# A Role for CD154, the CD40 Ligand, in Granulomatous Inflammation

**DOI:** 10.1155/2017/2982879

**Published:** 2017-07-12

**Authors:** Julien Villeneuve, Alexis Desmoulière, Antoine Dewitte, Nelly Bordeau, Pierre Costet, Laia Bassaganyas, Jean-Christophe Fricain, Jean Ripoche, Sébastien Lepreux

**Affiliations:** ^1^Cell and Developmental Biology Department, Centre for Genomic Regulation (CRG), The Barcelona Institute for Science and Technology, 08003 Barcelona, Spain; ^2^EA 6309 and Department of Physiology, Faculty of Pharmacy, University of Limoges, 87000 Limoges, France; ^3^Service d'Anesthésie-Réanimation II, CHU de Bordeaux, 33000 Bordeaux, France; ^4^Service des Animaleries, Université de Bordeaux, 33000 Bordeaux, France; ^5^Cardiovascular Research Institute and Institute for Human Genetics, University of California, San Francisco, CA, USA; ^6^INSERM U1026, Université de Bordeaux, 33000 Bordeaux, France; ^7^Pathology Department, CHU de Bordeaux, 33000 Bordeaux, France

## Abstract

Granulomatous inflammation is a distinctive form of chronic inflammation in which predominant cells include macrophages, epithelioid cells, and multinucleated giant cells. Mechanisms regulating granulomatous inflammation remain ill-understood. CD154, the ligand of CD40, is a key mediator of inflammation. CD154 confers a proinflammatory phenotype to macrophages and controls several macrophagic functions. Here, we studied the contribution of CD154 in a mouse model of toxic liver injury with carbon tetrachloride and a model of absorbable suture graft. In both models, granulomas are triggered in response to endogenous persistent liver calcified necrotic lesions or by grafted sutures. CD154-deficient mice showed delayed clearance of carbon tetrachloride-induced liver calcified necrotic lesions and impaired progression of suture-induced granuloma. In vitro, CD154 stimulated phagocytosis of opsonized erythrocytes by macrophages, suggesting a potential mechanism for the altered granulomatous inflammation in CD154KO mice. These results suggest that CD154 may contribute to the natural history of granulomatous inflammation.

## 1. Introduction

The clearance of undesirable material is a required checkpoint in the cascade of events that follows tissue injury and ends in the resolution of inflammation and tissue repair [1, 2]. This clearance step allows the removal of signals that would otherwise perpetuate inflammation. Granulomatous inflammation is a distinctive pattern of chronic inflammation in response to various infectious agents, such as mycobacteria or fungi, to noninfectious agents, as exemplified in the foreign body reaction (FBR) to biomaterials and in conditions from unknown causes including vasculitis, sarcoidosis, and Wegener's granulomatosis; predominant cells in granulomatous inflammation include macrophages, epithelioid cells, and multinucleated giant cells (MGCs) [3–15]. MGCs are macrophage polykaryons resulting from macrophage fusion [16]. Mechanisms responsible for MGC generation, as well as the functional significance of MGCs, remain incompletely understood [7, 17]. Apart from macrophages and MGCs, other cells, including T cells and fibroblasts, contribute to the progression of infectious or noninfectious granulomatous inflammation. A complex set of mediators is produced during the different phases of granulomatous inflammation, but a clear understanding of how these mediators orchestrate granulomatous progression is lacking. Cytokines, including interleukin- (IL-) 4, IL-12, IL-13, and interferon- (IFN-) *γ*, as well as matrix proteins and matrix remodeling proteins, including vitronectin, osteopontin, fibronectin, or matrix metalloproteinase- (MMP-) 9, have been shown to contribute [8, 18–28]. Angiogenesis is also a key event in granulomatous inflammation, and angiogenesis regulators are important contributors [29, 30]. The FBR is critical in biomaterial research as it negatively influences the integration of implanted devices and against which various strategies are actively sought for [12, 13, 31–36].

Platelet-derived mediators are likely to be involved in granulomatous inflammation. Indeed, the integral role of platelets in controlling several facets of the innate immune response is now being fully appreciated [37]. Activated platelets release a large array of inflammatory and mitogenic bioactive mediators with pleiotropic effects on target cells, including chemotaxis, adhesion, cell survival, and proliferation [38–41]. Platelets also stand at the cross-road of several proteolytic cascades linked with inflammation, such as coagulation and complement cascades [42]. Platelets are activated at sites of tissue injury, and implanted foreign materials activate platelets. However, mechanisms remain to be fully understood [12, 43, 44]. Among platelet mediators, CD154, a member of the tumor necrosis factor superfamily, may play a role in granulomatous inflammation. Indeed, the interaction between CD154 and its receptor CD40 controls a wide range of immune and inflammatory responses [40, 45–48]. CD40 is widely distributed, and mononuclear phagocytes express CD40 [49–52]. CD40 ligation by CD154 has consequences on inflammation, coagulation, extracellular matrix remodeling, metabolism, cell growth, and survival [45, 46]. Activated CD4^+^ T cells, activated platelets, and various other cells can be the source of CD154 [46]. Platelets represent a primary reservoir of CD154 in the blood [53–55].

CD40 triggering on macrophages has a profound effect on their biology, directing them towards an activated phenotype. CD40 signals macrophage functions for a large part towards expression of genes involved in proinflammatory response and tissue remodeling [52]. CD40 triggering noticeably induces the expression of a range of cytokines and chemokines, tissue factor, and MMPs, including MMP-1, MMP-3, MMP-9, MMP-11, and MMP-12, by macrophages and enhances IFN*γ*-induced production of nitric oxide [45, 51, 52, 56–64]. Accordingly, T cell-mediated activation of macrophages to produce nitric oxide or cytokines is impaired in the CD154-deficient mouse [57]. Therefore, CD40 signaling could be foreseen to control either directly or indirectly the macrophagic response in granulomatous inflammation.

Here, we studied the role of CD154 in granulomatous inflammation in two models of noninfectious granulomatous inflammation, a model of carbon tetrachloride- (CCl_4_-) induced liver injury and a model of absorbable suture graft. In these models, a granulomatous inflammation is triggered, in response to persistent liver calcified necrotic lesions or in response to the grafted biomaterial. We studied the progression of the granulomatous inflammation in wild-type (WT) and CD154-deficient mice (CD154KO). We also analyzed the role of CD154 on macrophage phagocytosis in vitro.

## 2. Material and Methods

### 2.1. Mice

Male Balb/cByJ CD154KO mice were generated from male Bl6/C CD154KO (B6.129S2-CD40lg^tm1Imx^/J) mice (Jackson Laboratory, ME) by repeated (>10) backcrossings. Animals were housed in a temperature-controlled pathogen-free environment (transgenic animal housing of Bordeaux University) with a 12 h light/dark cycle and given free access to food and water. The study followed the guidelines of the animal research ethical committee of Aquitaine Poitou-Charentes. Comparative blood counts showed no significant differences between WT and CD154KO animals (Supplementary experimental procedures available online at https://doi.org/10.1155/2017/2982879).

### 2.2. Carbon Tetrachloride Model

A dose of 300 *μ*L/kg body weight CCl_4_ (6% (*v*/*v*) solution in olive oil (olive oil for human consumption Puget®, France)) was injected intraperitoneally three times a week [65]. Eight mice were used at time 0, 4 at 1 injection, 4 at 2 injections, 8 at 3 injections, and 8 at 9 injections, for each group of mice.

### 2.3. Suture Bundle Grafts

Bundles of ten purple absorbable polyglactin 910 suture threads (Vicryl 0, Ethicon, Issy-les-Moulineaux, France), a synthetic poly(D,L-lactide-co-glycolide) (PLGA) copolymer [66–68], were implanted in the subcutaneous tissues on each mouse back; to track the implantation site, one thread of black nonabsorbable polyamid suture (Monosoft 0, Syneture, Covidien, France) was added within the bundles (Supplementary Figure 1). In experiments comparing WT and CD154KO mice, a total of 6 bundles were processed for each group.

### 2.4. Histology and Immunohistochemistry

CCl_4_ experiments: mice were sacrificed 48 hours after 1, 2, 3, or 9 CCl_4_ intraperitoneal injections. Liver samples were harvested, rapidly sliced to obtain sections from several lobes, and fixed in 3.7% formaldehyde. Paraffin-embedded liver sections (5 *μ*m) were stained with hematoxylin-eosin (HE) to evidence necrosis and cellular inflammation or with von Kossa stain to evidence calcifications. Immunohistochemical staining was performed with a rat anti-mouse F4/80 (AbD Serotec, UK). After heat-induced antigen retrieval (in citrate buffer, pH 6), endogenous peroxidases were inhibited. Then, liver sections were incubated with primary antibodies at a 1/100 dilution, for 1 hour at room temperature. The signal was amplified by the EnVisionTM dextran polymer (Dako A/S, Trappes, France) and revealed with liquid diaminobenzidine substrate (Dako A/S) before counterstaining with hematoxylin.

Suture bundle experiments: animals were sacrificed at week intervals. Because suture contained a nonabsorbable thread, at each time point of the experiment, the implantation sites on the back of the mice can be localized and removed en bloc. They were fixed in 3.7% formaldehyde, divided in four parts perpendicularly to the axis of the residual nonabsorbable thread, and paraffin embedded. Five serial sections (3 *μ*m) were performed on each paraffin-embedded bloc (Supplementary Figure 1B). Areas containing knots were excluded from the quantification. Each slide was stained with Masson's trichrome staining. With this staining, extracellular matrix is stained in green and the cytoplasm of the cells is stained in purple. Taking advantage of the contrast provided by this staining between the granuloma and the extracellular matrix, the granulomatous lesions of FBR can be easily outlined on the numerical microphotography taken with a camera-equipped light microscope and the area was quantified using the calibrated NIS-Elements Imaging software (Nikon France).

### 2.5. Liver Enzyme Measurements

Blood samples were collected from each animal by vein puncture before sacrifice. Sera were stored at −80°C until measurements of albumin, bilirubin, alkaline phosphatase (ALP), gamma-glutamyltransferase (*γ*-GT), aspartate aminotransferase (ASAT), and alanine aminotransferase (ALAT) on an automated analyzer in the Biochemistry Department of Pellegrin Hospital, Bordeaux, France.

### 2.6. Macrophages and Multinucleated Giant Cells

Macrophages and MGCs were obtained in vitro, as previously described [69]. Briefly, human mononuclear cells were obtained from platelet/leukocyte concentrates from healthy donors (convention with Etablissement Français du Sang Aquitaine-Limousin, Bordeaux, France) by centrifugation on a Ficoll gradient (Ficoll-Paque PLUS, GE Healthcare Life Sciences) and were suspended in complete medium Dulbecco's modified Eagle's medium supplemented with 10% heat-inactivated fetal calf serum (Perbio Science, Cramlington, UK), 2 mM L-glutamine (Invitrogen, Cergy Pontoise, France), 100 IU/mL penicillin (Invitrogen), and 100 *μ*g/mL streptomycin (Invitrogen). Cells were seeded in Primaria® 96-well tissue culture plates (VWR, Strasbourg, France) at 2.5 × 10^5^ cells per well in 100 *μ*L of culture medium. After 1 hour, nonadherent cells were removed by five washings; wells were then added with medium supplemented with granulocyte macrophage colony-stimulating factor (GM-CSF) (100 ng/mL) (Immunotools, Friesoythe, Germany) and the culture was maintained for two weeks. MGCs were generated by IL-4-dependent macrophage fusion: medium supplemented with GM-CSF (100 ng/mL) and IL-4 (100 ng/mL) (Immunotools) was added at day 7, and the culture was maintained for 15 additional days.

### 2.7. Real-Time RT-PCR Analysis

Total RNA was extracted from 50 mg of mouse frozen liver using a RNA extraction kit (NucleoSpin® RNAII Macherey-Nagel, Hoerdt, France) following the manufacturer's instructions and quantified spectrophotometrically (Thermo Spectronic, Cambridge, UK) from absorbance at 260 nm. To ascertain the purity of the extracted RNA, the 260 nm/280 nm ratio was measured, and the 18S and 28S components were visualized on agarose gel electrophoresis. Complementary DNA (cDNA) was synthesized with oligo-dT from 2 *μ*g of total RNA in a final volume of 40 *μ*L using First Strand cDNA Synthesis Kit for RT-PCR (Roche Applied Sciences, Meylan, France) according to the manufacturer's instructions.

The quantitative PCR was performed in triplicate on a Mx4000TM Multiplex Quantitative PCR System (Stratagene, Amsterdam, Netherlands) using iQ™ TMSYBR® Green Supermix kit (Bio-rad, Marnes la Coquette, France). The cycling parameters for quantitative PCR reaction included 40 cycles of denaturation at 95°C for 30 seconds, annealing at 62°C for 60 seconds, and elongation at 72°C for 30 seconds. The specificity of quantitative PCR was established by incorporating no reverse transcribed RNA. The specificities of the amplified transcripts were confirmed by melting curve profiles generated at the end of the PCR program and by sequencing. Mouse CD40 primers were as previously described [70]. Oligonucleotides used in this study are described in Supplementary experimental procedures. Ribosomal phosphoprotein P0 was used as a housekeeping gene standard.

### 2.8. Immunofluorescence

Cells were fixed by adding an equal volume of 2% paraformaldehyde (PFA). After 30 min at room temperature, Triton X-100 (0.1% *v*/*v*) was added for 5 min. Fixed and permeabilized cells were washed 3 times in phosphate-buffered saline, pH 7.2 (PBS), blocked 45 min in PBS/1% bovine serum albumin (BSA) (Sigma Aldrich, France), and incubated for 1 hour with an anti-human CD40 mouse antibody (mAb89, a kind gift from Dr. J. Banchereau, Dallas) in a humidified chamber. Slides were washed 3 times in PBS/1% BSA and incubated for 1 hour with a cross-adsorbed Alexa Fluor 488 goat anti-mouse IgG (H + L) (Interchim, Montluçon, France) at a 1/200 dilution in PBS/1% BSA. 4′,6′ Diamidino-2-phenylindole (DAPI) (Merck, Darmstadt, Germany) was used to stain nuclei and mounted with Vectashield (Vector Laboratories Inc., Burlingame, CA). Negative controls included indirect labeling with a primary isotype-matched mouse IgG followed by secondary antibody. Samples were visualized using a Leica SP5 confocal microscope (Leica Microsystèmes SAS, Rueil Malmaison, France).

### 2.9. Phagocytosis Assay

Macrophage and MGC phagocytosis of opsonized erythrocytes were assayed using the CytoSelect 96-well phagocytosis assay (Euromedex, Mundolsheim, France) following manufacturer's instructions. Briefly, cells were incubated with or without recombinant soluble CD154 (rsCD154) (MegaCD40LTM, Coger SAS, Paris, France) at 200 ng/mL, 24 hours before the addition of IgG opsonized sheep erythrocytes. Supernatants were aspirated after 15, 30, 60, and 120 min, and cells were washed with PBS to remove nonphagocytized erythrocytes. Adherent cells were then lysed, and the amount of phagocytized material was quantitated by a colorimetric assay. Negative control cells were treated with 2 *μ*M cytochalasin D to block phagocytosis.

### 2.10. Tartrate-Resistant Acid Phosphatase Activity Detection

Acid phosphatase activity in the presence of 50 mM tartrate was assayed with naphtol AS-MX phosphate (Sigma) as a substrate and freshly prepared Fast Violet B (Sigma). Liver sections were incubated overnight at 4°C in the substrate solution and counterstained with von Kossa. Cells were considered positive when evidencing strong pink staining with numerous red granules spread throughout the cytoplasm.

### 2.11. Statistics

Data are presented as means ± SD. Statistical comparisons between groups were performed using the Wilcoxon signed rank test. *p* < 0.05 was taken to imply statistical significance.

## 3. Results

### 3.1. CD154 Is Required for the Clearance of Liver Calcified Necrotic Lesions in Carbon Tetrachloride-Treated Mice

**Figure 1:**
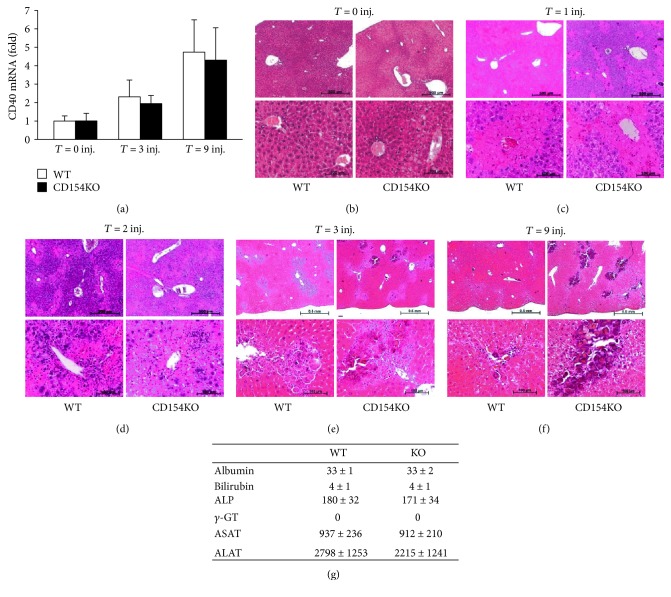
Carbon tetrachloride liver injury progression in WT and CD154KO mice. (a) Relative levels of CD40 mRNA expression (arbitrary units) in the liver of WT and CD154KO mice at time 0 and after 3 and 9 intraperitoneal CCl_4_ injections (mean ± SD, *n* = 8). Representative HE-stained liver tissue sections of WT and CD154KO mice at time 0 (b) and after 1 (c), 2 (d), 3 (e), and 9 (f) intraperitoneal CCl_4_ injections (inj.). Top panels, low magnifications (scale bar 500 *μ*m); bottom panels, high magnifications (scale bar 100 *μ*m); (*n* = 8 at time 0, *n* = 4 at 1 injection, *n* = 4 at 2 injections, *n* = 8 at 3 injections, and *n* = 8 at 9 injections for each group of mice). (g) WT and CD154KO mice show similar hepatic cytolytic response after CCl_4_ administration. Serum measurements of albumin (gL), bilirubin (*μ*mol/L), and liver enzymes (IU/L) after 1 CCl_4_ injection in WT and CD154KO mice (mean ± SD, *n* = 5).

**Figure 2:**
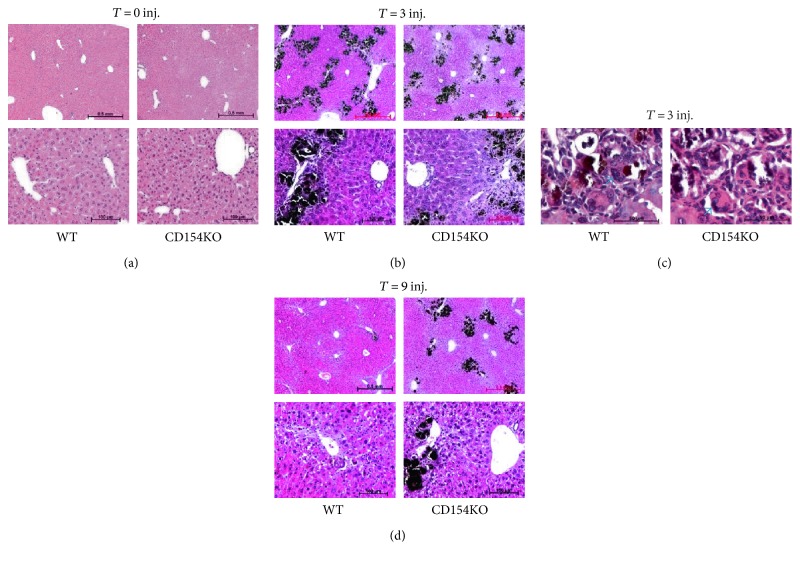
Differential clearance of dystrophic calcified necrotic liver lesions in WT and CD154KO mice: von Kossa staining. Representative von Kossa-stained liver tissue sections of WT and CD154KO mice at time 0 (a) and after 3 (b and c) and 9 (d) CCl_4_ injections (inj.). Top panels, low magnifications (scale bar 500 *μ*m); bottom panels, high magnifications (scale bar 100 *μ*m); (*n* = 8 for each condition in each group of mice). (c) High magnifications (scale bar 50 *μ*m) from (b) (*T* = 3 injections), highlighting MGCs associated to dystrophic calcified areas (arrows).

**Figure 3:**
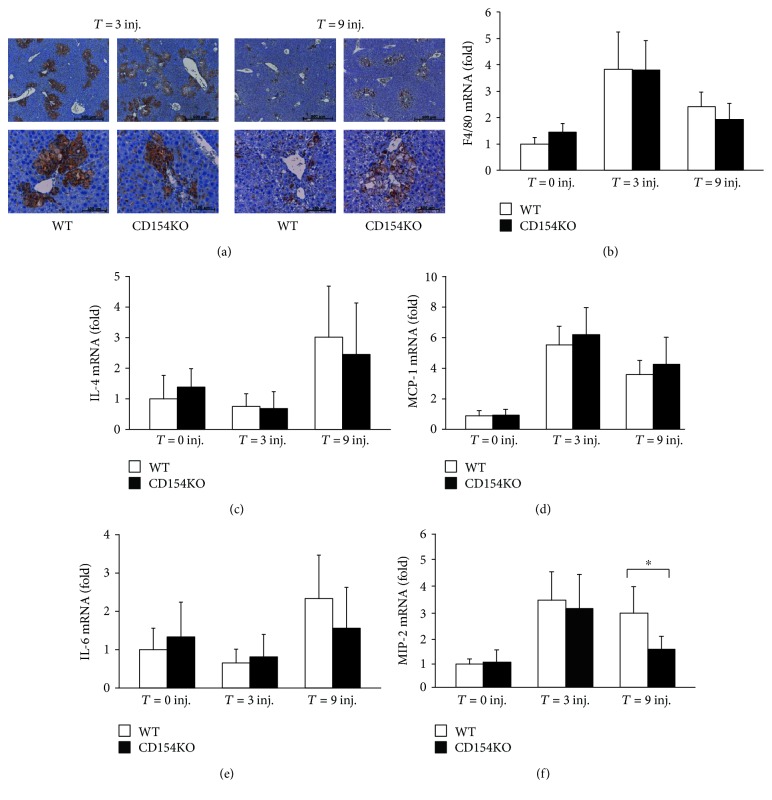
Immunohistochemical staining of monocyte/macrophage cells and cytokine/chemokine expression in WT and CD154KO mice after CCl_4_ injections. (a) Representative liver tissue sections from WT and CD154KO mice stained with the anti-F4/80 antigen monoclonal antibody after 3 and 9 CCl_4_ injections (inj.). Top panels, low magnifications (scale bar 500 *μ*m); bottom panels, high magnifications (scale bar 100 *μ*m) (*n* = 8 for each condition in each group of mice). Relative liver levels of F4/80 (b), IL-4 (c), MCP-1 (d), IL-6 (e), and MIP-2 (f) mRNA (fold arbitrary units), in WT and CD154KO mice at time 0 and after 3 and 9 CCl_4_ injections (inj.) (mean ± SD, ^∗^*p* < 0.05, *n* = 8).

To investigate the contribution of CD154 in granulomatous inflammation, we studied a murine model of endogenous lesions in WT and CD154KO mice. We used a murine model of liver calcified necrotic lesions induced by CCl_4_ administration. The CCl_4_ model has proven useful for the study of liver repair mechanisms; deficiencies in c-met signaling, plasminogen, and urokinase-type plasminogen activators lead to an impaired liver repair characterized by a delayed resolution of the granulomas around dystrophic calcified areas [71–73]. Hepatic expression of CD40 and liver histology did not differ between both mouse strains (Figures 1(a) and 1(b)). Parenchymal centrilobular necrosis was observed following the first CCl_4_ injection; the extent and the topographic distribution of necrotic lesions were similar in both strains (Figure 1(c)). Plasma liver enzyme measurements confirmed ongoing cytolysis and a similar magnitude of the liver injury between both mouse strains (Figure 1(g)). Following the second CCl_4_ injection, the general pattern of liver parenchymal necrosis remained unchanged but necrotic areas were infiltrated by inflammatory cells. There were no morphological differences in the inflammatory cell recruitment between both strains (Figure 1(d)). After three CCl_4_ injections, necrotic lesions, with apparent basophilic amorphous granular deposits of dystrophic calcifications surrounded by granulomas, were discernible in centrilobular regions of both strains (Figure 1(e)). Von Kossa staining confirmed calcium deposition within necrotic areas in both mouse strains (Figures 2(a), 2(b), and 2(c)); granulomas consisted of macrophages and MGCs, distributed throughout calcification (Figure 2(c)). The magnitude of parenchymal lesions was similar after three CCl_4_ injections; however, there was a divergence after nine CCl_4_ injections, with reference to the extent of dystrophic calcified necrotic areas. They persisted in CD154KO mice, whereas they had almost disappeared in WT mice (Figures 1(f) and 2(d)). Macrophages and MGCs associated to calcifications expressed the F4/80 antigen (Figure 3(a)). The quantification of F4/80 messenger RNA expression suggested similar macrophagic cell recruitment following CCl_4_-induced liver injury (Figure 3(b)). As IL-4 participates to foreign body giant cell generation in vivo, and as it is induced by CD154, we measured IL-4 expression by qRT-PCR in the livers of WT and CD154KO mice following CCl_4_ administration. IL-4 expression increased after nine CCl_4_ injections, but there were no differences between both strains (Figure 3(c)). MCP-1 has also been implicated as promoting macrophage fusion; its expression did not show differences between WT and CD154KO mice following CCl_4_ treatment (Figure 3(d)). The expression of other cytokines tested, IL-6 and MIP-2, also increased during the course of the CCl_4_ administration but there were no significant differences between WT and CD154KO, except for MIP-2 that showed reduction after 9 injections (Figures 3(e) and 3(f)). Finally, as osteoclasts also result from monocyte fusion and are involved in the resorption of calcified bone matrix, we also excluded the possibility that MGC associated with the dystrophic calcified areas could be osteoclasts by the absence of tartrate-resistant acid phosphatase activity; further, the expression of mRNAs for the receptor activator of NF-kB (RANK) and its ligand, a crucial dyad for the induction of osteoclasts, were not induced following CCl_4_ administration as well as for the expression of osteoprotegerin mRNA, a competitive RANK inhibitor (data not shown). Altogether, these results suggested impediment in the clearance of calcified necrotic lesions in CD154KO mice.

### 3.2. The Resolution of the Foreign Body Granuloma Is Altered in CD154KO Mice

**Figure 4:**
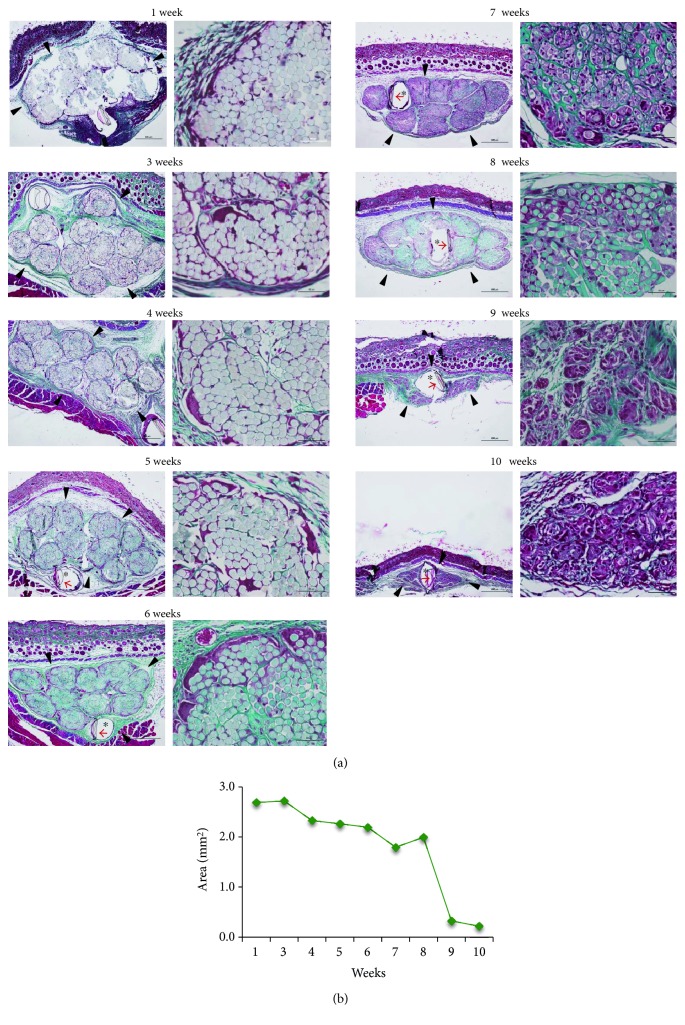
Absorbable suture bundles undergo a rapid bioresorption between weeks 8 and 10 postimplantation. (a) Tissue sections (low and high magnifications, scale bars 500 and 50 *μ*m for left and right panels, resp.) were obtained at week intervals and stained with Masson's trichrome. Microphotographs shown correspond to a representative kinetic of 3 experiments performed. Bundle contours showing rapid shrinking by week 8 are indicated by arrowheads. Red arrows indicate the nonabsorbable thread which, upon section by the microtome, creates an artefactual void on the tissue section (asterisk); (b) quantification of (a) suture bundle bioresorption.

**Figure 5:**
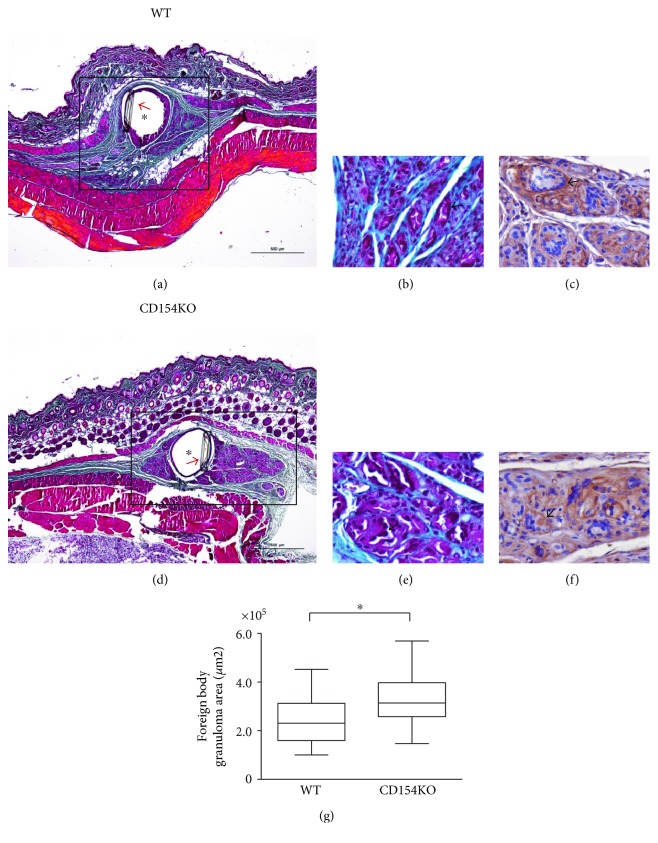
Differential clearance of suture bundles in WT and CD154KO mice. Representative Masson's trichrome-stained tissue sections from WT and CD154KO mice at week 10. (a and d) Low magnification (scale bar 500 *μ*m); arrows indicate the nonresorbable thread which, upon section by the microtome, creates an artefactual void on the tissue section (asterisk); squares highlight the FBR in subcutaneous tissues. (b and e) High magnifications (scale bar 20 *μ*m); the granulomas are composed of macrophages and multinucleated giant cells (MGCs) (arrows) bordered by fibrosis (green/blue). (c and f) Representative liver tissue sections from WT and CD154KO mice stained with the anti-F4/80 antigen monoclonal antibody at week 10; high magnifications (scale bar 20 *μ*m), macrophages, and MGCs (arrows) express the F4/80 antigen macrophagic marker. (g) Quantification of granuloma areas in WT and CD154KO mice (^∗^*p* < 0.05).

In an effort to strengthen these results, we developed a model of granuloma generated against implanted foreign material, a PLGA suture graft (Supplementary Figure 1 and Figure 4). Sutures represent one if not the most widely used biomaterial. Copolymers between glycolic acid and lactic acid have found large medical applications in surgical implants and also in biopolymer research as they represent promising scaffolds for cell support in tissue-engineering or drug-delivery devices [74]. Suture bundles were resorbed between weeks 8 and 10 postimplantation; the kinetic of resorption followed a sharp sigmoid inflection between weeks 8 and 10 postimplantation, after a lag phase of 6 weeks during which no significant variations of the bundle sizes were apparent (Figure 4). Morphologically, the host reaction had the form of typical granulomas, as expected [66–68]. After an initial acute inflammatory step, suture threads were progressively outlined by connective tissue and colonized by macrophagic cells. The FBR was characterized by granulomas containing numerous mononuclear macrophages, some MGCs and sparse lymphocytes. Between weeks 8 and 10, suture threads were cleared, leaving the nonabsorbable thread in the center of a residual granuloma at week 10 (Figure 4). Thus, week 10 was used as time point to compare the resorption of suture bundles between WT and CD154KO mice. Skin histology did not differ between both mouse strains. In both mouse strains, granulomas contained macrophages and MGCs as revealed by F4/80 staining (Figures 5(a), 5(b), 5(c), 5(d), 5(e), and 5(f)). However, the FBR area was larger in CD154KO mice compared to WT mice (Figure 5(g)). These results suggested an altered clearance of sutures bundles in CD154KO mice.

### 3.3. CD154 Stimulates Phagocytosis by Macrophages

**Figure 6:**
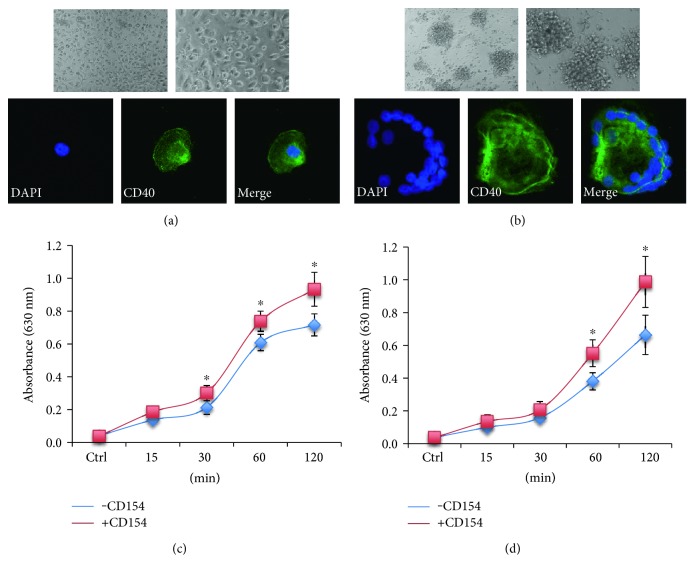
CD154 enhances phagocytosis by in vitro-derived macrophages. (a and b) CD40 is expressed by in vitro-derived macrophages and multinucleated giant cells (MGCs). (a) Macrophages derived from peripheral blood monocytes were immunostained for CD40 expression. Top panel, representative microphotographies of in vitro-derived macrophages (×100 and ×200 magnifications); bottom panel, CD40 immunostaining; nuclei were counterstained with DAPI (×400 magnification). (b) MGCs were obtained by IL-4-induced macrophage fusion and immunostained for CD40 expression. Top panel, representative microphotographies of in vitro-derived MGCs (×100 and ×200 magnifications); bottom panel, CD40 immunostaining; nuclei were counterstained with DAPI (×400 magnification). (c and d) CD154 enhances phagocytosis by in vitro-derived macrophages. (c) Cultures of in vitro-derived macrophages were assayed for phagocytosis in the presence or not of rsCD154. (d) Results obtained after fusion induction by IL-4. Cells were prestimulated 24 hours with 200 ng/mL rsCD154, and phagocytosis of opsonized erythrocytes was measured at various time-points (mean ± SD, *n* = 6; ^∗^*p* < 0.05).

Macrophage recruitment, adhesion to necrotic/calcified or foreign material, activation, and fusion of macrophages are key steps in granulomatous inflammation and FBR [12]. Signals that control macrophage phenotype and function are numerous, cytokines playing an important role. As CD40 triggering in macrophages leads to the induction of a pleiotropic range of effectors, including cytokines/chemokines and adhesion molecules, it could be hypothesized that CD154 interferes at various steps in granulomatous inflammation. First, we reexamined CD40 expression on macrophages and on MGCs. MGCs were generated by IL-4-mediated fusion [69]. In the absence of IL-4, macrophagic cells remained scattered; upon IL-4 treatment, they fused together forming MGCs harboring nucleus numbers which ranged from 20 to 40 admixed with scattered remaining macrophages; both macrophages and IL-4-induced MGCs expressed CD40 (Figures 6(a) and 6(b)). The addition of rsCD154 neither increased the frequency nor the size of MGCs, nor altered cell morphology, suggesting that it had no effect on the cell fusion process (data not shown). As essential key step in the resolution of the inflammatory reaction is the phagocytic clearance by macrophagic cells, we investigated whether CD154 has a regulatory action on phagocytosis. As expected [75], isolated human macrophages ingested IgG-opsonized erythrocytes. The presence of rsCD154 in cultured macrophages resulted in increased phagocytosis of opsonized red cells (Figure 6(c)). Phagocytosis of IgG-opsonized erythrocytes was also observed in cultures associating MGCs, and macrophages after IL-4-mediated fusion and rsCD154 had a stimulatory effect (Figure 6(d)), a situation that would more closely represent the admixed MGCs and macrophages granuloma cell population. However, as cultures of MGCs also include scattered macrophages, whether CD154 stimulates MGC phagocytosis needs confirmation in isolated MGC cultures.

## 4. Discussion

Although conditions leading to granulomatous inflammation are diverse, there are common histologic features and mechanisms in granuloma generation. An intricate array of cell and biological mediators coordinate in an ill-defined way the progression of granulomatous inflammation [4, 10]. In this work, we show that, in two different models of noninfectious granuloma, the absence of CD154 is associated with an altered granulomatous inflammation progression.

CD154 may play a role in granulomatous inflammation by regulating macrophage functions. Indeed, macrophages are key immune cell effectors in granulomatous inflammation. Resident macrophages or macrophages derived from recruited monocytes are subjected to a range of signals at sites of granulomatous inflammation that activate them and induce differential functional programs. Macrophages are involved in virtually every step of granulomatous inflammation, orchestrating the proinflammatory granuloma microenvironment and being critical in fueling chronic inflammation and subsequent fibrosis [3, 5, 10, 12, 13, 76–78].

Macrophages express CD40, and the binding of CD154 activates monocytes/macrophages with essential consequences in tissue homeostasis by driving cytokine/chemokine, protease production and control of tissue clearance capacity, pathogen killing, or anti-tumor effects [51, 56, 58, 79–82]. The absence of CD154 is therefore likely to impact macrophage activation [51, 57]. As there was a lack of evident differences in the extent of the initial lesions, inflammatory cell recruitment, and extent of MGC generation, we hypothesize that the impaired clearance of the necrotic and calcified tissue and of suture bundles could be partly dependent on a phagocytosis defect. First, phagocytosis is an important mean through which macrophages contribute to tissue homeostasis, including the clearance of apoptotic/necrotic cells and damaged tissue components [83–85], and phagocytosis is regulated by the CD40 signaling ([86] and our study). Macrophage phagocytosis impairment is associated to delayed tissue repair [87, 88]. Our results suggested that CD154 has a regulatory role on macrophage phagocytosis. Second, the absence of CD154 can be expected to reduce cytokine production by macrophages. Hence, CD154 deficiency, through the impairment of macrophage activation, may alter the transition towards the resolution of granulomatous inflammation. Indeed, the early stages of inflammation, including macrophage clearance of apoptotic cells, determine following resolution stages [1]. Macrophages are a heterogeneous population [89]. M1 macrophages mostly contribute to inflammation while M2 macrophages mostly contribute to tissue repair and remodeling. However, being not fully distinctive, CD40 is highly expressed on M1 macrophages relatively to M2 macrophages [90, 91]. Differently polarized macrophages coexist in FBR as shown for example by in situ studies [92]. Biomaterials have also been shown to induce a shift in M1/M2 polarization dependent on the nature of the material [78, 93, 94], and M1/M2 polarization may be linked to different outcome in granuloma progression [95, 96]. Macrophage polarization can be regulated by the CD40 signaling. For example, IFN-*γ* in combination with CD154 can switch M2 to M1-like macrophages [97, 98] and macrophages of mice deficient in CD40-TRAF6 signaling show a polarization towards a M2 signature [99]. However, macrophages present in the FBR may not always fit with the classical M1/M2 phenotype signature [100] and the assignment of macrophages to a particular M1 or M2 lineage in granulomatous inflammation remains an open question needing studies of dynamic changes in macrophage phenotype transition in the progression of granulomatous inflammation. The role of CD154 in M1/M2 transition and how this could affect progression of granulomatous inflammation therefore remains to be analyzed.

The role of CD154 on functional properties of MGCs will require experiments with purified MGC populations. Apart from their role on the degradation/resorption of the foreign material [101], MGCs are thought to be instrumental in the phagocytic clearance of foreign material but little is known about the magnitude of their contribution and how this function is regulated. Indeed, human MGCs derived from cultured macrophages have phagocytic properties [75]. MGCs are also the source of inflammatory mediators and interestingly adapt the pattern of inflammatory mediators they produce depending on the nature of the foreign material they encounter [102].

Control of macrophage activation by CD154 does not exclude other potential mechanisms for the altered granulomatous inflammation in the absence of CD154. Additional mechanisms may be related to the regulation of the global cytokine network by CD154, as, in addition to macrophages, CD154 is a general cytokine/chemokine inducer for a variety of cells. Various cytokine deficiencies are indeed associated to altered granuloma progression [14, 103]. The absence of CD154 is likely to alter the cytokine network in the granuloma milieu. Among cytokine expression that we studied in the liver injury model, MIP-2 was found to be downregulated; MIP-2 is produced by a variety of cells, including macrophages, in response to injury and is an important mediator of inflammation in acute liver injury [104]. The downregulation of MIP-2 expression in CD154-deficient mice suggests potential involvement of MIP-2 in the progression of granuloma inflammation.

Another point of discussion is the source of CD154 during the progression of granulomatous inflammation. Activated platelets are a major source of CD154 in the organism. Together with macrophages and other inflammatory cells, platelets are immediately available and activated at sites of tissue injury and likely to play an important role in providing inflammatory mediators, including CD154. Platelets are important players in the immune system. Platelet-associated and soluble CD154 are important contributors to the inflammatory reaction [45, 46], and the role of platelets in initiating, sustaining, and resolving inflammation is increasingly stressed [37]. It is tempting to speculate that they contribute to the natural history of granulomatous inflammation. However, potential other sources of CD154 exist, as various cells, including CD4^+^ T cells and fibroblasts, that are important cell components and functional players in granuloma progression [105]. In inflammatory conditions, these cells can both express CD154 at their surface and release a soluble form. Therefore, the granuloma inflammatory milieu is likely to be a source of CD154. Apart from mechanisms underlying CD154 contribution, the control of the foreign body reaction in order to slow or prevent the formation of a fibrotic capsule remains a major challenge in the field of biomaterial research. The responsibility of macrophages in the fibrotic process is well established, and the CD40/CD154 dyad may represent a potential target to delay fibrotic encapsulation of implanted materials. Altogether, our study suggests that CD154 contributes to the natural history of granulomatous inflammation.

## Supplementary Material

Supplementary information: Supplementary experimental procedure 1: Mice. Supplementary experimental procedure 2: Oligonucleotide primers used in Real Time RT-PCR analysis. Supplementary figure 1. Suture bundle implantation protocol.
