# Supplementary material for: A Role for CD154, the CD40 Ligand, in Granulomatous Inflammation
Source: Mediators Inflamm. 2017 Jul 12;2017:2982879. doi: 10.1155/2017/2982879 (PMC5529663; doi:10.1155/2017/2982879)
Supplement: Supplementary file 1 — Supplementary information: Supplementary experimental procedure 1: Mice. Supplementary experimental procedure 2: Oligonucleotide primers used in Real Time RT-PCR analysis. Supplementary figure 1. Suture bundle implantation protocol. [file 2982879.f1.pdf]

Supplementary information: 2 supplementary experimental procedures and 1 supplementary figure

Mice

CD154KO mice: comparative blood counts showed no differences between WT and CD154KO animals except for a discrete lymphocytopenia in CD154KO animals that did not reach statistical significance (4.22±1.26 $10^3/mm^3$ versus 5.8±2.22 $10^3/mm^3$ in CD154KO and WT animals, respectively, n=6). There were no differences in the platelet counts of WT and CD154KO mice (1242±179 $10^3/mm^3$ and 1160±74 $10^3/mm^3$, respectively, n=6). Mice were free of mouse hepatitis virus as determined by qRT-PCR (QM Diagnostics, Nijmegen, The Netherlands).

Oligonucleotide primers used in Real Time RT-PCR analysis

Oligonucleotide Name	Sequence (5'-3')
mRPLO-Fw	CGCGACCTGGAAGTCCAACT
mRPLO-Rev	CCATCAGCACCACAGCCTTC
mCD40-Fw	GGGTGGCATTGGGTCTTCTC
mCD40-Rev	GGTCCATCTAGGGCAGTGTG
mF4/80-Fw	GCTAGTGGAGGCAGTGATGC
mF4/80-Rev	CAGGACTGGAAGCCCCATAGC
mIL4-Fw	CCCCCAGCTAGTTGTCATCC
mIL4-Rev	CGTCCCTTCTCCTGTGACCT
mIL6-Fw	CCTTCTTGGGACTGATGCTG
mIL6-Rev	CCTCCGACTTGTGAAGTGGT
mMCP1-Fw	TTCTGGGCCTGCTGTTCAC
mMCP1-Rev	ACACCTGCTGCTGGTGATC
mMIP2-Fw	GCTGTTGTGGCCAGTGAAC
mMIP2-Rev	GCCCTTGAGAGTGGCTATGA
mOsteoprotegerin-Fw	CGGAGAGTGAGGCAGGCTAT
mOsteoprotegerin-Rev	TGCTCAGCTGTGAGGAGAGG
mRANK-Fw	CTCGGTGAGGTCTTGGGAAC
mRANK-Rev	TAAGCTGCCTATGGGATGGAC

mRANKL-Fw	AGGGAGCACGAAAAACTGGTC
mRANKL-Rev	GGAAGGGTTGGACACCTGAAT

mRPLO, murine ribosomal phosphoprotein P0; mMCP1, murine monocyte chemoattractant protein 1; mIL4 and 6, murine interleukin-4 and -6; mMIP2, murine macrophage inflammatory protein 2; mRANK , murine receptor activator of nuclear factor kappaB; mRANKL, murine receptor activator of nuclear factor kappaB ligand.

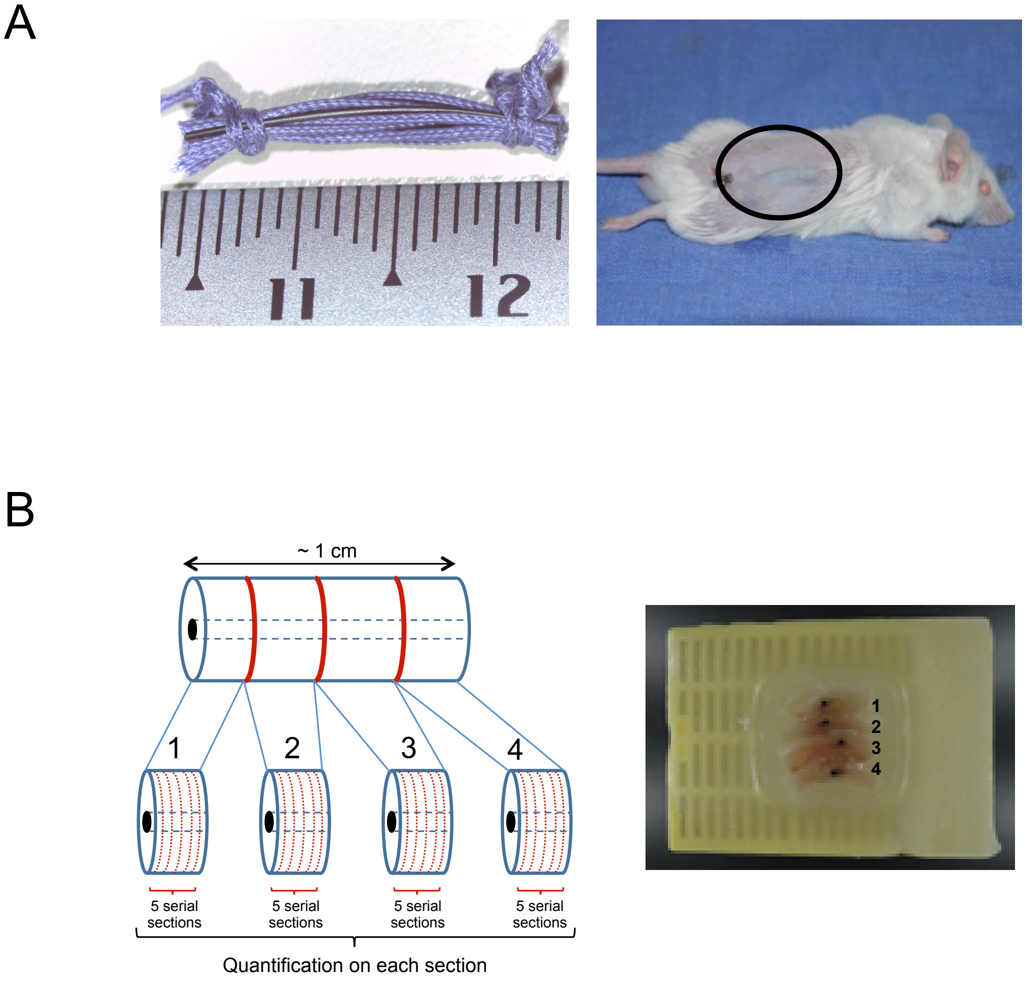

Supplementary figure 1. Suture bundle implantation protocol.
(A) Left panel depicts photograph of a bundle highlighting the nonaborbable thread used to track the implantation site; right panel depicts how suture bundles were implanted on mouse backs. (B) Processing of suture bundle implants for histological analysis. Implantation sites on mouse backs were localized with the nonabsorbable thread and largely excised en bloc. They were then immediately fixed with formalin solution, divided in four parts perpendicularly to the axis of the non-absorbable thread and paraffin embedded. Five serial sections were performed on each paraffin-embedded bloc, stained with Masson's trichrome and processed for histological analysis.
